# ^18^F-fluorodeoxyglucose positron emission tomography-positive sarcoidosis after chemoradiotherapy for Hodgkin’s disease: a case report

**DOI:** 10.1186/1752-1947-5-247

**Published:** 2011-06-29

**Authors:** Martin H Cherk, Alan Pham, Andrew Haydon

**Affiliations:** 1 Department of Nuclear Medicine, The Alfred Hospital, Commercial Road, Melbourne, Victoria 3004, Australia; 2 Department of Anatomical Pathology, The Alfred Hospital, Commercial Road, Melbourne, Victoria 3004, Australia; 3 Department of Medical Oncology, The Alfred Hospital, Commercial Road, Melbourne, Victoria 3004, Australia

## Abstract

**Introduction:**

The occurrence of granulomatous disease in the setting of Hodgkin's disease is rare; however, when it occurs it can pose significant clinical and diagnostic challenges for physicians treating these patients.

**Case presentation:**

We report the case of a 33-year-old Caucasian woman of Mediterranean descent with newly diagnosed ^18^F-fluorodeoxyglucose (^18^F-FDG) positron emission tomography (PET)/computed tomography (CT) scan-positive, early-stage Hodgkin's disease involving the cervical nodes who, despite having an excellent clinical response to chemotherapy, had a persistent ^18^F-FDG PET scan-positive study, which was suggestive of residual or progressive disease. A subsequent biopsy of her post-chemotherapy PET-positive nodes demonstrated sarcoidosis with no evidence of Hodgkin's disease.

**Conclusion:**

This case highlights the fact that abnormalities observed on posttherapy PET/CT scans in patients with Hodgkin's disease are not always due to residual or progressive disease. An association between Hodgkin's disease and/or its treatment with an increased incidence of granulomatous disease appears to exist. Certain patterns of ^18^F-FDG uptake observed on PET/CT scans may suggest other pathologies, such as granulomatous inflammation, and because of the significant differences in prognosis and management, clinicians should maintain a low threshold of confidence for basing their diagnosis on histopathological evaluations when PET/CT results appear to be incongruent with the patient's clinical response.

## Introduction

The use of positron emission tomography (PET)/computed tomography (CT) to evaluate lymphoma, including Hodgkin's disease, continues to increase worldwide and is considered the standard of care when available for pretreatment staging and assessment of treatment response.

PET has been demonstrated to modify disease stage (usually upstage) in about 15% to 20% of patients and affect therapeutic management in about 5% to 15% of patients [1]. PET is considered significantly more accurate than CT for the assessment of treatment response because of its ability to distinguish between metabolically active tumor or fibrosis in posttherapy residual masses, which are present in approximately two-thirds of patients with Hodgkin's disease.

Although PET/CT is a highly sensitive technique for detecting Hodgkin's disease, other conditions, such as infection, inflammation and granulomatous disease, may result in a false-positive scan. An increased incidence of granulomatous disease has been associated with Hodgkin's disease and/or its treatment [2-4]; hence a positive posttherapy PET/CT scan needs to be interpreted with caution, particularly if it is incongruent with the patient's clinical response.

Here we present the case of a patient with residual PET scan positivity following seemingly successful treatment of early-stage Hodgkin's disease which subsequently was confirmed to be due to granulomatous disease.

## Case presentation

**Figure 1:**
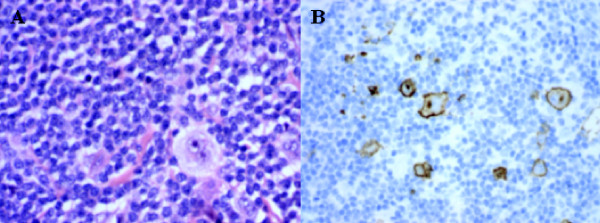
**Biopsies confirming nodular sclerosing Hodgkin's lymphoma with aggregates of small lymphocytes interspersed with CD30+ Reed-Sternberg cells**. **(A) **Hematoxylin and eosin stain; original magnification, × 400. **(B) **CD30+ cells. Original magnification, × 400.

**Figure 2:**
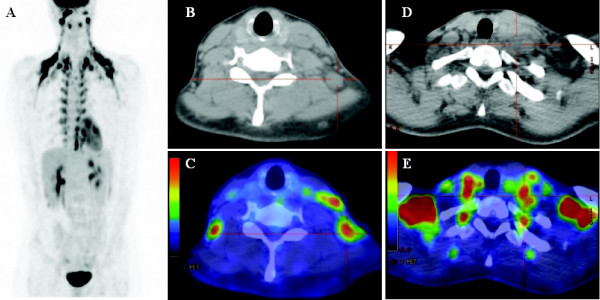
**Pretreatment ^18^F-fluorodeoxyglucose (^18^F-FDG) positron emission tomography/computed tomography scan**. **(A) **Prominent physiological brown fat uptake in the neck and thorax. **(B and C) **^18^F-FDG-avid lymphadenopathy in the left lower cervical nodes. **(D and E) **^18^F-FDG-avid lymphadenopathy in the left supraclavicular regions.

A 33-year-old, previously well Caucasian woman of Mediterranean descent presented to our hospital with asymptomatic left cervical lymphadenopathy for further investigation. There were no associated symptoms of night sweats, weight loss or fever. A subsequent excisional biopsy of a left supraclavicular node demonstrated features consistent with classical nodular sclerosing Hodgkin's lymphoma, with nodular aggregates of small lymphocytes interspersed with CD30+ and CD15+ lacunar variants of Reed-Sternberg cells (Figure 1). A pretreatment staging ^18^F-fluorodeoxyglucose (^18^F-FDG) PET/CT scan was confounded by prominent physiological brown fat uptake in the neck and thorax, but confirmed ^18^F-FDG-avid lymphadenopathy in the left lower cervical and left supraclavicular regions with no evidence of disease elsewhere in the body (PET scan-confirmed stage IIA Hodgkin's lymphoma) (Figure 2). Her bone marrow biopsy was negative for bone marrow involvement. She was subsequently treated with two cycles of ABVD (doxorubicin, bleomycin, vinblastine and dacarbazine) chemotherapy followed by involved field radiotherapy (30gy delivered in 17 fractions to the left upper chest and neck).

**Figure 3:**
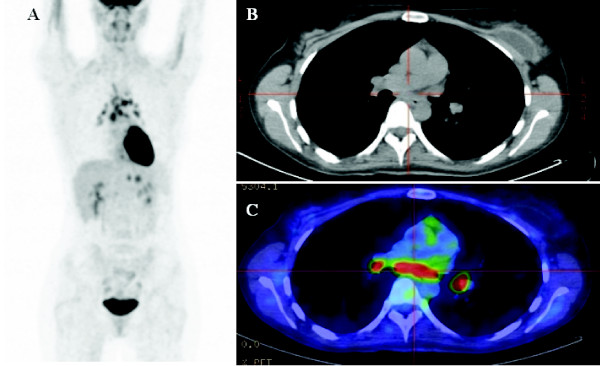
**Posttreatment ^18^F-fluorodeoxyglucose (^18^F-FDG) positron emission tomography/computed tomography scan showing resolution of ^18^F-FDG-avid lymphadenopathy in the left cervical and supraclavicular nodes but new bilateral ^18^F-FDG-avid pulmonary hilar and mediastinal lymphadenopathy**.

**Figure 4:**
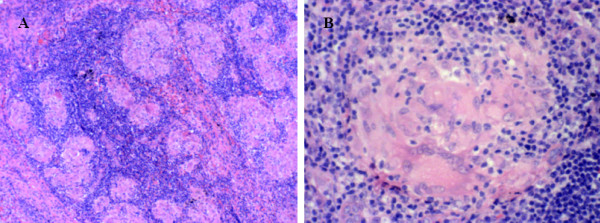
**Mediastinal nodal sampling showing non-necrotizing granulomatous inflammation with numerous multinucleated giant cells consistent with sarcoidosis**. **(A) **Hematoxylin and eosin stain; original magnification, × 100. **(B)** Hematoxylin and eosin stain; original magnification, × 400.

A posttherapy restaging ^18^F-FDG PET/CT scan was performed two months following the completion of radiotherapy. The scan demonstrated complete resolution of ^18^F-FDG-avid lymphadenopathy in the left cervical and supraclavicular regions, but new widespread ^18^F-FDG-avid bilateral pulmonary hilar and mediastinal lymphadenopathy, raising the possibility of progressive Hodgkin's disease (Figure 3). A mediastinoscopy was performed, and several mediastinal nodes were obtained for histopathological evaluation. These specimens demonstrated non-necrotizing granulomatous inflammation with numerous multinucleated giant cells, consistent with sarcoidosis (Figure 4). No evidence of malignancy was detected, and staining for infective organisms was negative.

Subsequent investigations, including serum and urinary calcium levels and an ophthalmological review for ocular sarcoidosis, were normal. Her pulmonary function tests demonstrated a mild decrease in gas transfer (62% of predicted value) which was nonspecific and possibly related to prior radiotherapy. Her chest X-ray was normal. Cardiac magnetic resonance imaging was performed to evaluate a left anterior hemiblock pattern visualized on her electrocardiogram, which was negative for cardiac sarcoidosis. A nonsteroidal anti-inflammatory drug regimen was commenced for mild chest discomfort thought to be related to mediastinal lymphadenopathy, with good relief of symptoms. A course of corticosteroids was deemed unnecessary upon a respiratory physician's review, and the proposed management plan was one of close observation.

## Discussion

An association between sarcoidosis and Hodgkin's lymphoma has been raised previously in the literature, including several case reports and case series describing sarcoidosis preceding or occurring concurrently with Hodgkin's lymphoma [4,5]. An association of sarcoidosis and lymphoma has also been described by Brincker, who found that patients with sarcoidosis have a five and one-half times higher incidence of developing lymphoma associated with their sarcoidosis [2]. Sporadic case reports have described sarcoidosis following treatment of Hodgkin's lymphoma [3,6,7].

Sarcoidosis is a chronic multisystem disorder of unknown cause that typically affects young to middle-aged adults and causes non-necrotizing granulomas composed of epithelioid histocytes which replace the normal lymph node architecture and at times also infiltrate body organs such as the lungs. The prevalence of sarcoidosis is estimated to be between 10 to 20 per 100,000 population; however, the prevalence is not known with certainty and can vary greatly between geographical regions. Sarcoidosis is three to four times more common among African-Americans, who have a lifetime risk of 2.4% compared to a 0.85% risk among the Caucasian population in North America [8,9].

Interestingly, sarcoidosis and Hodgkin's disease appear to have many immunological features in common, such as cutaneous anergy, peripheral lymphopenia and prominent infiltration of helper T cells at sites of involvement. It is possible that a similar underlying dysregulation in immune function or an immunosuppressive state predisposes individuals to both conditions.

Among the other hypotheses that have been postulated is that Hodgkin's disease itself or its treatment may contribute to the development of sarcoidosis through immunosuppression or other mechanisms, such as an idiosyncratic drug reaction. ABVD therapy is a commonly utilized regimen for the treatment of Hodgkin's disease. Bleomycin differs pharmacokinetically from other drugs in ABVD combination therapy and has a relatively higher tissue concentration in the skin, lungs, kidneys, peritoneum and lymphatics relative to hematopoietic tissues [6]. Interestingly, sarcoidosis has a predilection for similar organs; hence a relationship between the two has been raised [6].

^18^F-FDG PET/CT is considered the standard tool for primary staging and assessment of treatment response in patients with Hodgkin's lymphoma because of its high overall sensitivity (> 95%) and specificity (> 90% pretreatment and 70% to 80% posttreatment) for detecting lymphoma [10-12]. False-positive results do occur, however, particularly following treatment (in up to 10% to 40% of cases) [12,13], as a result of other concomitant conditions, such as infection, inflammation and granulomatous disease, all of which have the potential to cause increased ^18^F-FDG uptake during PET.

^18^F-FDG PET has been demonstrated to have high sensitivity (78% to 95%) for detecting granulomatous changes and inflammation, with evidence in the literature confirming its utility as a noninvasive means of monitoring treatment response in patients with sarcoidosis. In many centers, ^18^F-FDG PET has surpassed the traditionally used gallium-67 scan for the diagnosis and management of patients with sarcoidosis because of its higher sensitivity for detecting active sites of disease [14].

It is likely that an increasing number of cases of sarcoidosis will be detected in association with Hodgkin's lymphoma or its treatment because of the increasing use of ^18^F-FDG PET as the main imaging modality to stage and assess the progress of this disease. Although biopsy and histopathological evaluation comprise the only definitive technique to confirm granulomatous disease and exclude residual or recurrent lymphoma, clinical features and certain patterns of ^18^F-FDG uptake on PET scans often suggest an alternate diagnosis, such as granulomatous disease.

Bilateral pulmonary hilar and mediastinal nodal ^18^F-FDG uptake particularly, if not in an original site of disease on pretreatment PET scans, should raise the possibility of an alternate pathology to Hodgkin's lymphoma following chemotherapy. This is even more true in patients with early-stage Hodgkin's lymphoma, such as in the present case, in which the prognosis would be expected to be excellent and relapse or progressive disease following first-line chemotherapy would be considered unusual. Symmetry is an important diagnostic feature of metabolically active pulmonary hilar and mediastinal lymphadenopathy associated with sarcoidosis, which often differentiates it from alternate pathologies, such as lymphoma, on PET scans [15].

The lungs are the most common extranodal site of involvement with sarcoidosis, and changes can often be seen on CT scans, particularly if CT is performed at high resolution [16]. Small, 1 mm to 5 mm pulmonary nodules with irregular borders predominantly found in the upper and middle lung zone are the most common and almost universal finding where there is pulmonary involvement [17]. The presence of architectural distortion associated with these nodules is a key feature of pulmonary sarcoidosis, which helps to differentiate it from lymphoma [18]. Pulmonary fibrosis occurs in 20% to 25% of patients with pulmonary sarcoidosis [15]; however, this usually takes years to develop and is not a particularly specific finding in Hodgkin's disease patients treated with bleomycin, as this drug itself is associated with pulmonary fibrosis.

Extrathoracic involvement can also occur with sarcoidosis and warrants consideration in situations in which the PET/CT findings are incongruent with the patient's clinical response. Examples of extrathoracic sites of disease include any nodal group within the body, skin, eyes, liver, spleen, muscles, bones and parotid glands.

Apart from Hodgkin's disease, granulomatous inflammation has also been associated with other neoplasms, such as T-cell lymphomas, seminomas of the testis, renal cell carcinoma and nasopharyngeal carcinoma, where the presence of granulomas in the tumor parenchyma has been attributed to the cytokine milieu of either the main tumor of other cells comprising the tumor background [19]. It is therefore of vital importance to scrutinize granulomas closely on the basis of histological examinations in the setting of neoplasia to avoid the underdiagnosis of residual viable malignancy.

Differential diagnoses for granulomatous inflammation in patients with a history of neoplasia include foreign body reaction to necrotic tumors, associated systemic illness such as tuberculosis caused by immunosuppression or a direct reaction to one of the therapeutic agents given to the patient [19].

## Conclusion

Abnormalities on PET/CT scans in patients treated for Hodgkin's disease are not always due to residual or progressive lymphoma, and clinicians should retain a high index of suspicion for alternative pathologies when PET/CT results appear incongruent with the patient's clinical presentation or response to therapy. An association appears to exist between Hodgkin's disease and/or its treatment with an increased incidence of granulomatous disease. Certain patterns of ^18^F-FDG uptake on PET scans may suggest granulomatous disease rather than lymphoma, and because of the differences in the prognosis and management of the two conditions, clinicians should maintain a low threshold for biopsy and histopathological correlation in these situations.

## Consent

Written informed consent was obtained from the patient for publication of this case report and any accompanying images. A copy of the written consent is available for review by the Editor-in-Chief of this journal.

## Competing interests

The authors declare that they have no competing interests.

## Authors' contributions

MC was responsible for the acquisition of data, analyzing and providing the PET/CT scan images for the figures and drafting the manuscript. AP was responsible for providing anatomical pathology images for the figures, scientific revision of the report and discussion and editing of the manuscript. AH was responsible for the clinical management of the patient, scientific revision of the report and discussion and editing of the manuscript. All authors read and approved the final manuscript.
